# Cellular Recovery and Body Composition Changes in Pediatric Celiac Disease After the Start of a Gluten-Free Diet: A Prospective Cohort Study

**DOI:** 10.3390/jcm14145061

**Published:** 2025-07-17

**Authors:** Teresa Nestares, María Jiménez-Muñoz, Encarnación Torcuato-Rubio, Laura Tamayo Pérez, Marta de la Flor Alemany, Marta Herrador-López, Víctor Navas-López, Rafael Martín-Masot

**Affiliations:** 1 Department of Physiology, Faculty of Pharmacy, University of Granada, 52001 Granada, Spain; nestares@ugr.es (T.N.); ltamayoperez@correo.ugr.es (L.T.P.); rafammgr@gmail.com (R.M.-M.); 2 Institute of Nutrition and Food Technology “José Mataix Verdú” (INYTA), Biomedical Research Centre (CIBM), University of Granada, 52001 Granada, Spain; 3 Pediatric Unit, Hospital la Serranía, 29400 Ronda, Spain; mariajmnzm@gmail.com; 4 Pediatric Gastroenterology and Nutrition Unit, Hospital Regional Universitario de Málaga, 29010 Málaga, Spain; encatr@gmail.com (E.T.-R.); herradorlopezm@gmail.com (M.H.-L.); 5 Department of Nutrition and Food Science, Campus of Melilla, University of Granada, 52001 Melilla, Spain; floralemany@ugr.es; 6 Department of Pharmacology and Pediatrics, School of Medicine, University of Málaga, 29071 Málaga, Spain

**Keywords:** celiac disease, gluten-free diet, children, body composition, bioelectrical impedance analysis

## Abstract

**Background/Objectives:** Celiac disease (CD) alters nutrient absorption and body composition, especially during childhood. Although adherence to a gluten-free diet (GFD) promotes mucosal recovery, its impact on cellular functionality and metabolic balance remains underexplored. This study aims to evaluate the utility of bioelectrical impedance vector analysis (BIVA) in assessing nutritional status, inflammatory improvement, and body composition changes in pediatric patients with CD following a GFD. **Methods**: Seventy-nine children aged 5–14 years were studied. Three groups were analyzed: (1) 25 children with newly diagnosed CD, evaluated at diagnosis and after 12 months of GFD (prospective cohort); (2) 25 CD patients on a GFD for over 24 months (cross-sectional); and (3) 29 healthy controls. Body composition (fat mass (FM), fat-free mass (FFM), body cell mass (BCM), phase angle (PhA), and Na^+^/K^+^ ratio) was measured. GFD adherence was assessed and a dietary assessment was also performed. **Results**: After 12 months on a GFD, newly diagnosed CD patients showed significant increases in FM (from 8.2 to 10.1 kg, *p* = 0.001), FFM (*p* = 0.001), and BCM (*p* = 0.0001), along with a significant decrease in the Na^+^/K^+^ ratio (*p* = 0.015). Compared to healthy controls, CD children on GFD for more than 24 months had higher FM (12.2 vs. 8.8 kg, *p* = 0.013) and lower Na^+^/K^+^ ratios (*p* = 0.006). PhA increased slightly over time but did not reach statistical significance. **Conclusions**: Our study suggests that the adherence to a GFD leads to improved body composition and cellular homeostasis in children with CD, as reflected by increases in BCM and reductions in Na^+^/K^+^ ratio, making it a promising biomarker for monitoring inflammation and cellular recovery.

## 1. Introduction

Celiac disease (CD) is a systemic immune-mediated disorder triggered by the ingestion of gluten and related prolamins present in cereals such as wheat, barley and rye, in genetically predisposed individuals [1]. It affects approximately 1–1.4% of the population; however, despite advances in diagnostic techniques, a substantial proportion of cases remain undiagnosed, particularly during critical periods of development such as childhood, increasing the risk of adverse health outcomes [2,3].

The only available treatment for CD is a strict gluten-free diet (GFD). At diagnosis, some children with CD may still present with nutritional deficiencies and altered body composition, including reduced bone mineral density, largely attributable to malabsorption from villous atrophy and persistent intestinal inflammation [4,5,6]. However, it is increasingly common to observe non-classical or even asymptomatic presentations, in which subtle nutritional alterations may still be present. Although adherence to a GFD generally promotes mucosal recovery and restores adequate nutrient absorption within a few months [7,8,9,10], its impact on body composition raises concerns. Gluten-free products often contain higher amounts of carbohydrates and fats, contributing to an increased prevalence of overweight and obesity, which has been reported in up to 21% of patients, both at diagnosis and during follow-up [11]. Moreover, previous studies have documented dietary imbalances following the initiation of the GFD, including excessive or deficient nutrient intake patterns that could further influence body composition and metabolic health [12,13].

Given the risk of altered body composition in children with CD, anthropometric monitoring becomes a key element in clinical follow-up. Regular assessment of parameters such as weight, height, body mass index (BMI) and bone mineral content provides valuable information about growth trajectories and nutritional recovery following GFD initiation. Previous studies consistently report that children with CD tend to present with lower body weight, lean mass and bone Z-scores compared to healthy counterparts at diagnosis [14,15]. These deficits often improve following sustained adherence to a well-balanced GFD, which can even result in compensatory growth in some cases [16,17]. Nonetheless, conventional anthropometric measures may not fully capture subtle changes in body composition related to inflammation and malabsorption. In line with this approach, current ESPGHAN guidelines [4] recommend regular monitoring of nutritional status in pediatric CD patients, primarily through anthropometric parameters.

To overcome these limitations, complementary techniques such as bioelectrical impedance vector analysis (BIVA) are increasingly used to provide a more comprehensive evaluation of nutritional status and body composition. BIVA is a non-invasive, accessible and reproducible method that estimates fat-free mass (FFM) and hydration status based on electrical properties of body tissues [18,19]. Although traditional BIA approaches rely on predictive equations that may be limited in pathological conditions, phase-sensitive BIA (SF-BIA) offers additional raw parameters such as resistance (R) and reactance (Xc), from which the phase angle (PhA) is derived. PhA has emerged as a robust biomarker reflecting cellular integrity and hydration status and has demonstrated prognostic value in assessing nutritional and inflammatory states [20].

In the context of CD, its utility remains fully underexplored, though early studies suggest it may be a valuable tool for monitoring nutritional recovery during GFD implementation [21]. In fact, emerging evidence suggests that PhA could be a valuable tool in CD management. Studies in adults with CD have demonstrated lower PhA values at diagnosis compared to healthy individuals, correlating with malnutrition risk and altered fluid distribution [21]. Similar trends have been observed in pediatric populations, although the experience in children remains largely anecdotal, with improvements in PhA following dietary intervention being associated with mucosal recovery and enhanced nutrient absorption [16].

Since a GFD is the only effective treatment for CD and inflammation plays a key role both in the disease itself and in body composition, this study aimed to evaluate whether 12 months of adherence to a GFD led to significant improvements in anthropometric parameters, body composition and cellular recovery in children with CD, with a special emphasis on PhA and body cell mass (BCM) as markers of nutritional and inflammatory status.

## 2. Materials and Methods

### 2.1. Study Design and Participants

A total of 79 children aged 5–14 years were enrolled in the study. Participants were recruited between 31st May 2023 and November 2024, and CD diagnosis was made based on the criteria from the European Society of Pediatric Gastroenterology, Hepatology, and Nutrition (ESPGHAN) [1].

The study consisted of three groups: (1) a cohort of 25 children newly diagnosed with CD, before initiating a GFD, who were followed prospectively for 12 months and reevaluated at the end of the follow-up period; (2) a control group comprising 29 healthy children with a negative serological screening and no history of chronic disease who presented with minor symptoms related to chronic functional constipation, according to the Rome IV criteria [22], or other non-gastrointestinal issues; and (3) a group of 25 celiac patients who had adhered to a GFD for more than 24 months, evaluated at a single time point. Control patients were matched to cases by age and sex in order to minimize potential selection bias. This matching strategy was applied prior to the comparison of clinical and analytical variables between groups.

The inclusion criteria for the control group were age between 5 and 14 years, absence of serum IgA anti-transglutaminase (tTG) antibodies, normal weight for their age, no gastrointestinal disorders in the previous year and a normal appetite. Exclusion criteria for both groups included liver or kidney diseases, acute or chronic inflammation, inflammatory bowel disease, diabetes, chronic asthma and the use of dietary supplements with antioxidant properties. Additionally, patients with obesity (as defined by the International Task Force criteria) [23] and those refusing to sign the informed consent were excluded.

Written informed consent was obtained from all parents. The study was approved by the Ethics Committee (Ref. 1875-N-22) and was conducted in accordance with the principles of the Declaration of Helsinki and its subsequent amendments.

### 2.2. Clinical and Sociodemographic Characteristics

Participants’ clinical and sociodemographic characteristics were assessed by the same group of researchers.

### 2.3. Anthropometric Measures

Weight (kg) was assessed with a scale (InBody R20, Biospace, Seoul, Republic of Korea), and height (cm) was measured using a stadiometer (Seca 22, Hamburg, Germany). Height and weight were used to calculate the BMI (weight [Kg]/height [m^2^]).

The assessment of body composition was carried out using electrical bioimpedance with TANITA MC-980MA multifrequency equipment (Biológica Tecnología Médica S.L., Barcelona, Spain) and its integrated Biological Suite 7.1 software (version 1.4). The percentage values of FM, lean mass, muscle mass (MM), bone mass, total body water (TBW), basal metabolic rate and PhA were recorded.

### 2.4. Dietary Assessment

Dietary intake was assessed using a 24 h recall over three days, including one non-working day. Parents or guardians recorded the food and beverages consumed by the children, noting quantities and brands. Household measurements were converted to grams using an equivalence table. The data were analyzed using the Evalfinut 2.0 software, which includes the Spanish food composition database (BEDCA) [24] and the United States Department of Agriculture database (FoodData Central) [25]. Both were supplemented with nutritional information from gluten-free products. The program calculated energy intake, the proportion of macro- and micronutrients, and the adequacy of intake according to age, physical activity and BMI recommendations.

### 2.5. Evaluation of Gluten-Free Diet Adherence and Fecal Sample Analysis

Adherence to the GFD was assessed after four months of dietary intervention. At that time, participants were asked to provide two stool samples collected on non-consecutive days—one sample during a weekday and the other on a weekend day.

The concentration of gluten immunogenic peptides (GIPs) in stool samples was assessed using a lateral flow assay (iVYCHECK GIP Stool kit, Biomedal S.L., Seville, Spain) according to the manufacturer’s instructions. This assay is a rapid immunochromatographic test designed to qualitatively detect the presence of GIPs in fecal samples, yielding either a positive or negative result. Previous studies have reported that this method achieves a sensitivity between 95% and 100% and a specificity of 100% [26,27].

### 2.6. Statistical Analyses

Variables with a normal distribution were expressed as mean ±standard deviation, and those without it were expressed as median and interquartile range (IQR). The Kolmogorov–Smirnov test was used to assess the normality of the distribution. The chi-square test was used to compare proportions. Student’s *t*-test was used to compare variables with a normal distribution, and the Mann–Whitney U and Kruskal–Wallis tests were used for those without a normal distribution. A *p*-value < 0.05 was considered statistically significant. Data were analyzed using the SPSS^®^ statistical package, version 24.0 for MacOS^®^ (SPSS, Inc., Chicago, IL, USA).

As dietary intake variables—including energy, fat, protein, carbohydrates, sugar, and fiber—were not normally distributed, non-parametric tests were applied. Results are presented as median and IQR. Differences in dietary intake between the three independent groups (newly diagnosed celiac patients at baseline, celiac patients after two years on a gluten-free diet, and healthy controls) were evaluated using the Kruskal–Wallis test. Longitudinal changes in dietary intake within the celiac group (T0 vs. T12) were assessed using the Wilcoxon signed-rank test. A two-tailed *p*-value < 0.05 was considered statistically significant.

## 3. Results

Differences in body composition parameters between celiac patients at the onset of the disease, after two years on a GFD, and healthy controls are shown in Table 1. A significantly higher FM was observed in celiac patients after two years on a GFD compared to newly diagnosed patients and healthy controls (*p* = 0.048). No significant differences were found in other body composition parameters, including fat-free mass (FFM), fat mass index (FMI), or PhA (all *p* > 0.05). However, the Na^+^/K^+^ ratio was significantly lower in celiac patients after two years on a GFD compared to newly diagnosed patients and healthy controls (*p* = 0.037).

Differences in body composition parameters between celiac patients following a GFD for at least two years and the control group are presented in Table 2. Regarding body fat composition, celiac patients following a GFD for at least two years had significantly higher FM compared to controls (12.2 [7.8–16.1] kg vs. 8.8 [6.3–11.6] kg; *p* = 0.013), whereas FMI was higher but did not reach statistical significance (*p* = 0.061). Lean body mass parameters, including FFM (*p* = 0.133), FFMI (*p* = 0.322), BCM (*p* = 0.110), and body cell mass index (BCMI) (*p* = 0.149), showed no significant differences between groups. Muscle quality markers, including PhA (*p* = 0.170) and PhA Z-score (*p* = 0.158), were comparable between groups. However, Na^+^/K^+^ ratio was significantly lower in celiac patients on a GFD than in controls (*p* = 0.006).

**Figure 1:**
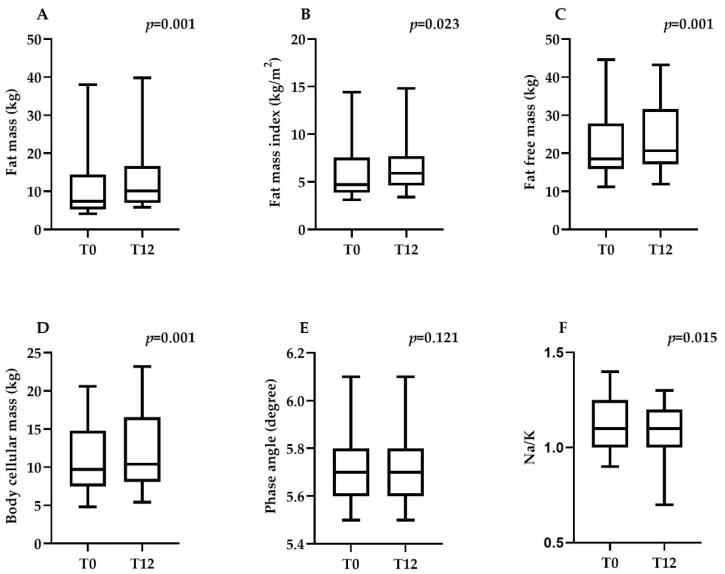
Differences in body composition parameters in patients with celiac disease at the onset of the disease (T0) and after 12 months (T12) following a gluten-free diet (*n* = 25). Box plots show the median, interquartile range (IQR), and whiskers representing the minimum and maximum values. (**A**) Differences in fat mass (kg) between T0 and T12. (**B**) Differences in fat mass index (kg/m^2^) between T0 and T12. (**C**) Differences in fat-free mass (kg) between T0 and T12. (**D**) Differences in body cellular mass (kg) between T0 and T12. (**E**) Differences in phase angle (degree) between T0 and T12. (**F**) Differences in Na^+^/K^+^ ratio between T0 and T12.

Differences in body composition parameters in patients with CD at the onset of the disease (T0) and after 12 months (T12) following a GFD are shown in Supplementary Table S1. No significant differences were observed in weight Z-score between T0 and T12 (*p* = 0.989), whereas height Z-score significantly decreased (*p* = 0.005). Additionally, BMI Z-score showed a significant increase over the 12-month period (*p* = 0.026). Regarding body fat composition, there was a significant increase in FM from 7.4 kg (5.2–14.4) at T0 to 10.1 kg (6.9–16.6) at T12 (*p* = 0.001), as well as an increase in FMI (*p* = 0.023). FFM increased significantly (*p* = 0.001), while FFMI remained stable (*p* = 0.397). Similarly, BCM showed a significant increase (*p* = 0.0001), whereas BCMI exhibited a non-significant trend toward improvement (*p* = 0.059). Regarding muscle quality, PhA and Z-score PhA showed slight, non-significant improvements (*p* = 0.121 and *p* = 0.128, respectively). Although PhA showed a slight improvement over 12 months of GFD, this change did not reach statistical significance, suggesting variability in individual recovery or a potential need for a longer follow-up period. However, the Na^+^/K^+^ ratio significantly decreased over the 12-month period (*p* = 0.015). These findings indicate that 12 months on a GFD are associated with significant increases in fat and lean body mass (Figure 1).

Differences in body composition parameters between celiac patients after 12 months on a GFD and healthy controls are shown in Supplementary Table S2. Celiac patients had a significantly higher FMI compared to healthy controls (*p* = 0.05). Regarding muscle quality, PhA was significantly higher in celiac patients after 12 months on a GFD (*p* = 0.026 and *p* = 0.049, respectively), and the Na^+^/K^+^ ratio was significantly lower in celiac patients compared to healthy controls (*p* = 0.035).

Table 3 presents the comparative analysis performed using the Kruskal–Wallis test, which revealed no statistically significant differences in dietary intake among newly diagnosed celiac patients at baseline, celiac patients after two years on a GFD, and healthy controls. Median energy intake was comparable across the three groups (1697.3 [1381.8–1972.1] kcal at baseline, 1837.1 [1457.5–2193.0] kcal after 2 years on GFD, and 1858.4 [1622.8–2037.4] kcal in controls; *p* = 0.889). Similarly, no significant differences were observed in fat, protein, carbohydrate, sugar, or fiber intake (all *p* > 0.05).

Table 4 presents the longitudinal comparison of dietary intake in celiac patients between baseline (T0) and after 12 months on a GFD (T12). Wilcoxon signed-rank tests showed no statistically significant changes in any dietary variable. Although median energy intake increased from 1697.3 [1381.8–1972.1] kcal to 2076.4 [1672.0–2232.7] kcal, the change was not statistically significant (*p* = 0.126). Similar non-significant increases were observed in protein, carbohydrate, sugar, and fiber intake (all *p* > 0.05).

## 4. Discussion

This study shows, through an anthropometric analysis based on BIVA, how the GFD, carried out correctly by celiac children, leads to improvements in parameters related to cellular integrity and inflammatory status (PhA and, fundamentally, Na^+^/K^+^ ratio), compared to controls, which may be attributed to the normalization of the inflammatory status. Equally, a strict GFD for 12 months in newly diagnosed celiac children has an impact on other body composition parameters, mainly BCM and FM. These changes may not necessarily reflect a recovery from a deteriorated state but rather the maintenance or optimization of nutritional status through improved nutrient absorption and inflammation control.

Currently, the long-term effects of the GFD on the cellular physiology and inflammatory status of celiac patients are still under investigation. In this context, the cellular Na^+^/K^+^ exchange has emerged as a valuable parameter. Na^+^/K^+^-ATPase is a crucial membrane-bound enzyme responsible for maintaining electrochemical gradients across the plasma membrane. Beyond its classic role in ion transport, it has been increasingly recognized as a sensitive marker and mediator of cellular stress in inflammatory conditions. Na^+^/K^+^-ATPase is highly sensitive to redox state alterations and undergoes reversible post-translational modifications such as S-glutathionylation, S-nitrosylation, and carbonylation under oxidative stress, leading to inhibition of its activity and increased degradation via proteasomal and lysosomal pathways. These disturbances promote sodium retention, cell swelling, metabolic dysfunction, and even apoptosis [28,29,30,31]. Evidence from renal and cardiovascular models shows that cytokine-mediated or ROS-induced inhibition of the pump can contribute to apoptosis and inflammation persistence [31,32] and may plausibly extend to the intestinal epithelium in pediatric CD. Interestingly, in CD, cytokines can promote oxidative stress by stimulating ROS and RNS production and thus may impair Na^+^/K^+^-ATPase function and stability through these redox-sensitive mechanisms described above [33,34,35,36]. Thus, dysfunction of the Na^+^/K^+^ pump may contribute to impaired cellular integrity, fluid imbalance, and chronic inflammation in this context. The Na^+^/K^+^ ratio, reflecting the functional status of this pump, has shown potential as a clinically relevant marker of inflammation, malnutrition, and overall cellular health [32,37]. In our study, this ratio decreased significantly in children with celiac disease after 12 months of GFD, suggesting not only nutritional recovery but also reversal of inflammation-induced pump dysfunction. Moreover, celiac children on a GFD for both 12 months and more than 2 years showed significantly lower Na^+^/K^+^ ratios compared to healthy controls, which may reflect a persistent adaptation of cellular physiology under dietary treatment, supporting the idea of anti-inflammatory effects of GFD and its role in the improvement of CD-associated cellular dysfunction.

Another important parameter in the assessment of nutritional status is BCM. The two components that make up body mass are FM and FFM. FFM includes skin, skeletal muscles, bone tissue, visceral organs and TBW (intra- and extracellular). Considering FFM without extracellular water and bone mineral mass results in BCM, which is the most metabolically active compartment of the body [38]. In our study, a significant improvement in BCM is observed after 12 months of GFD in celiac children (BCMI shows a tendency to improve, but not significantly). Therefore, its increase suggests that nutritional recovery in celiac patients is not only limited to normalization of weight or BMI but involves a recovery of metabolically active cell mass and thus of functionality. Similarly, Wiech et al. [39], in a case-–control study with celiac children, demonstrated that after one year of GFD, celiac children show significantly higher values of BCM, as well as FFM and MM. Maniero et al. [21], in a study carried out in newly diagnosed celiac adults, followed up for one year after the start of the GFD, and healthy controls, found a significant increase in BCMI, showing a gradual improvement, already present 6 months after the start of the diet. In this study, a significant difference in BCMI is also observed, lower in celiac patients at diagnosis than in controls. Kostecka et al. [16], in BIA analysis of body composition of adult celiac patients at diagnosis and 9 months after initiation of the GFD, found a significant increase in skeletal muscle mass (SMM), which is part of the BCM. Finally, Skoracka et al. [40] also found differences in MM and FFM in celiac women (significantly lower) compared to healthy women, without reference, in this case, to time or adherence to the GFD.

A parameter related to BCM is PhA, which is an index of cellular integrity and functionality that decreases in situations of inflammation, malnutrition and sarcopenia [20,41,42]; therefore, its value is especially important in the pediatric age group, due to the negative consequences of states of malnutrition and inflammation in this population. In our study, we observed a trend towards improvement of PhA in celiac children after 12 months of GFD, suggesting a progressive recovery of cellular nutritional status, reflecting improved cell membrane integrity and metabolic functionality. However, the lack of statistical significance indicates that this recovery, although clinically relevant, could be heterogeneous between patients or require a longer period to reach significance. This reinforces the need for larger cohorts or longer-term monitoring to validate this trend as clinically meaningful. Individual factors such as adherence to the GFD, diet quality, residual inflammatory status or the time required for intestinal mucosal regeneration could influence the magnitude of improvement. Other studies, carried out in adults, have found a significant increase in PhA in celiac patients after the establishment of a GFD, such as the aforementioned work by Maniero et al., which demonstrates a significant increase in PhA in celiac adults 6 months and 12 months after the onset of GFD, as well as a significantly lower PhA at diagnosis compared to healthy controls [21]. Similarly, Kostecka et al. also found a significant increase in PhA in adults with CD 9 months after the establishment of the GFD [32]. The non-significance found in our study may be explained by the differential factors inherent to CD in children and adults, such as the lower frequency of extradigestive symptoms in children [43], which may lead to a later diagnosis and, therefore, a slower recovery of anthropometric parameters. Therefore, further studies are needed in this area, particularly in the pediatric population.

Notably, our study reveals that the PhA values of celiac children after more than 24 months of PDG are equal to those of the control group, suggesting that strict long-term GFD not only reverses malabsorption, but also allows a progressive and complete recovery of cellular functionality and homeostasis and the nutritional and metabolic state of the organism. This could also be related to a reduction in oxidative stress and chronic inflammation, essential aspects in the pathogenesis of CD [36].

Finally, we found a significant increase in FM and FMI in celiac children after 12 months of GPD, with the latter group also having significantly higher FMI values compared to healthy controls. Similarly, significantly higher FM values are observed in celiac children after 2 years of GFD compared to healthy controls. Previous reviews of studies including both adults and children [44] find that patients not treated with GFD have lower FM values compared to control groups, with this relationship being more disparate between treated celiac patients (with no indication of GFD compliance time) and control groups. The increase in FM and FMI in celiac children after the onset of GFD may be due to nutritional recovery, with changes in body composition by improving intestinal absorption and decreasing inflammatory status and, therefore, energy expenditure due to the disease, but it may also be due to the higher sugar and fat content of ultra-processed products frequently used in GFDs [12]. These results align with recent meta-analyses exploring body composition in celiac patients before and after GFD. Vereczkei et al. found a significant increase in FM after one year of GFD, although treated celiac patients still showed lower FM and FFM than non-celiac controls, suggesting an incomplete normalization of body composition despite dietary adherence [45]. In contrast, Xin et al. observed that while GFD did not significantly impact FM overall, subgroup analyses showed increased FM and BMI in celiac patients, particularly with interventions exceeding 48 weeks, highlighting that prolonged dietary adherence may promote fat gain due to both improved absorption and the nutritional composition of gluten-free products [46]. This increase in FM, even compared with healthy controls, may be a sign of adequate nutritional recovery if it occurs in proportion to the increase in FFM and BCM, but it may also carry a potential risk of overweight, obesity, and long-term metabolic complications, especially if the FM gain is disproportionate to MM. However, in our cohort, these changes cannot be attributed to differences in dietary intake, as no significant variations in dietary patterns were observed throughout follow-up. Therefore, it would be interesting to assess whether the sustained increase in FM is associated with insulin resistance, dyslipidemia, or inflammatory markers. While this increase may partly reflect nutritional recovery, disproportionate fat mass gain, especially in the absence of corresponding lean mass accrual, could represent an early sign of metabolic imbalance or increased long-term risk of sarcopenic obesity. In this regard, Yerushalmy-Feler et al. [47] conducted an observational study in celiac patients aged 5 to 20 years, where they analyzed the interaction between body composition and metabolic syndrome (there is no clear consensus on the definition of this syndrome in childhood). They found that individuals with components of metabolic syndrome (e.g., high diastolic blood pressure) had higher total and truncal body fat percentages (FATP and TFATP, respectively), as well as lower muscle-to-fat ratio (MFR) (indicative of sarcopenic obesity), regardless of weight status.

These findings reinforce the need for long-term nutritional follow-up in children with CD, not only to ensure adherence to the GFD, but also to assess diet quality and make recommendations regarding physical activity and dietary habits, with the aim of preventing a disproportionate FM gain. However, it should be noted that we found no significant differences in body composition parameters between newly diagnosed celiac patients and controls, which could be explained by the short diagnostic delay at our center. This lack of difference may be explained by early diagnosis and prompt dietary intervention, which could limit the extent of nutritional deterioration at baseline. However, it may also reflect a limitation in our sample size or study power, which should be addressed in future research. The results of other studies in celiac children do show that patients have significantly lower mean FM, FFM, and TBW values compared to healthy controls, although they include patients both at diagnosis and at different stages of treatment [29].

### Strengths and Limitations

While most nutritional studies on CD have focused on pediatric patients, there are few that analyze body composition using BIA, with most traditionally emphasizing BMI. A notable strength of our study is that we monitored not only anthropometric, body composition and bioelectric parameters, but also dietary intake, allowing us to rule out changes in diet as a confounding factor. This comprehensive approach, supported by the involvement of professional nutritionists throughout the follow-up, reinforces the reliability of our findings and provides a more robust understanding of the observed changes in body composition.

Regarding the limitations of our study, there are several that should be considered. Firstly, the patient sample is relatively small, so the results obtained should be interpreted with caution, and larger studies would be necessary. However, the strict inclusion criteria and the homogeneous, well-characterized cohort strengthen the internal validity and reproducibility of our findings. Secondly, the lack of follow-up beyond 12 months of GFD adherence prevents us from knowing the long-term evolution, which would be necessary to achieve possible statistical significance in parameters such as PhA. Although FM and BCM were reported as absolute values, the lack of significant differences in age and sex across groups supports the validity of between-group comparisons. These methodological considerations enhance the internal validity of our results. Thirdly, some of the observed trends did not reach statistical significance, possibly due to limited sample size, and should be interpreted cautiously in light of a potential type II error. Finally, longitudinal follow-up was not conducted in the control group, as no intervention was applied and the study design did not aim to assess changes over time in this population.

## 5. Conclusions

Our study suggests that the adherence to a GFD leads to progressive nutritional recovery or preservation and improved cellular functionality in celiac children, as evidenced by the increase in BCM and PhA and the decrease in Na^+^/K^+^ ratio. In this sense, the Na^+^/K^+^ ratio could represent a promising early biomarker of cellular recovery and systemic inflammation resolution in pediatric CD. However, some benefits may take longer to reach clinical relevance, highlighting the need for extended follow-up. Additionally, the observed increase in FM in celiac children following a GFD underscores the importance of tailored nutritional guidance and lifestyle recommendations to prevent excessive fat accumulation and its associated metabolic risks. BIVA emerges as a valuable tool for monitoring nutritional status over time, reinforcing the necessity of a comprehensive and individualized approach in the long-term management of celiac patients. Compared with conventional tools, BIVA is practical, non-invasive and inexpensive; its portable device can be used at the bedside, delivers results in under two minutes, and is well-tolerated by children, enabling repeated follow-up without radiation or discomfort. Although it provides no regional data and is somewhat sensitive to hydration status, these minor drawbacks are offset by its ease of use and everyday clinical utility. Future research should explore whether these improvements translate into enhanced physical performance, reduced fatigue, and better quality of life.

## Figures and Tables

**Table 1:** Differences in body composition parameters between celiac patients at different stages of their disease vs. healthy controls.

	Celiac Patients Basal (*n* = 25) ^1^	Celiac Patients 2y GFD (*n* = 25) ^2^	Healthy Controls (*n* = 29)	*p*
**Age (years)**	10 (8–12)	11 (8.5–12)	9 (7.5–11.5)	0.255
**Females, *n* (%)**	13 (52)	17 (68)	17 (58.6)	0.091
**Anthropometry**				
Z score Weight	0.1 (−0.8–0.6)	−0.1 (−0.6–0.7)	−0.02 (−1.7–0.6)	0.744
Z score Height	−0.4 (−1.1–0.4)	−0.3 (−0.9–0.9)	0.08 (−1.4–0.7)	0.561
Z score BMI	0.06 (−0.3–0.7)	0.1 (−0.43–1.2)	−0.2 (−1.2–0.7)	0.536
**Body Fat composition**				
FM (kg)	7.4 (5.2–15.6)	12.2 (7.8–16.1)	8.8 (6.3–11.6)	**0.048**
FMI (kg/m^2^)	4.7 (3.8–7.6)	6.0 (4.4–8.0)	5.0 (3.8–5.9)	0.180
**Body lean mass composition**				
FFM (kg)	18.5 (15.8–30.3)	25.9 (17.6–32.6)	20.5 (16.6–25.7)	0.230
FFMI (kg/m^2^)	11.7 (11.1–13.1)	12.4 (10.9–13.5)	11.5 (10.6–12.7)	0.597
BCM (kg)	9.7 (7.5–15.4)	12.5 (8.5–16.2)	9.7 (7.7–12.7)	0.236
BCMI (kg/m^2^)	5.8 (5.1–7.1)	6 (5.3–6.7)	5.6 (5.0–6.1)	0.346
**Muscle quality**				
PhA (degree)	5.2 (4.7–5.5)	5 (4.9–5.4)	5.0 (4.7–5.2)	0.298
Z score PhA	−1.1 (−2.1–−0.2)	−1.1 (−1.5–−0.8)	−1.3 (−1.9–−0.8)	0.342
Na^+^/K^+^ *	1.124 ± 0.1665	1.072 ± 0.1100	1.169 ± 0.1257	**0.037**

^1^ Celiac patients at diagnosis who had at least two visits during follow-up (baseline and 12 months). ^2^ Celiac patients with 2 years of GFD. Data are given as median (interquartile range). BCM, body cellular mass; BMI, body mass index; FFM, free fat mass; FFMI, fat mass index; FM, fat mass; FMI, fat mass index; PhA, phase angle. * The data are shown as mean (standard deviation).

**Table 2:** Differences in body composition parameters between celiac patients following a gluten-free diet for at least two years versus healthy controls.

	Celiac Patients 2y GFD (*n* = 25)	Healthy Controls (*n* = 29)	*p*
**Age (years)**	11 (8.5–12)	9 (7.5–11.5)	0.104
**Females, *n* (%)**	17 (68)	17 (58.6)	0.587
**Anthropometry**			
Z score Weight	−0.1 (−0.6–0.7)	−0.02 (−1.7–0.6)	0.482
Z score Height	−0.3 (−0.9–0.9)	0.08 (−1.4–0.7)	0.477
Z score BMI	0.1 (−0.43–1.2)	−0.2 (−1.2–0.7)	0.358
**Body Fat composition**			
FM (kg)	12.2 (7.8–16.1)	8.8 (6.3–11.6)	**0.013**
FMI (kg/m^2^)	6.0 (4.4–8.0)	5.0 (3.8–5.9)	0.061
**Body lean mass composition**			
FFM (kg)	25.9 (17.6–32.6)	20.5 (16.6–25.7)	0.133
FFMI (kg/m^2^)	12.4 (10.9–13.5)	11.5 (10.6–12.7)	0.322
BCM (kg)	12.5 (8.5–16.2)	9.7 (7.7–12.7)	0.110
BCMI (kg/m^2^)	6 (5.3–6.7)	5.6 (5.0–6.1)	0.149
**Muscle quality**			
PhA (degree)	5 (4.9–5.4)	5.0 (4.7–5.2)	0.170
Z score PhA	−1.1 (−1.5–−0.8)	−1.3 (−1.9–−0.8)	0.158
Na/K *	1.072 ± 0.1100	1.1669 ± 0.1692	**0.006**

Data are given as median (interquartile range). BCM, body cellular mass; BCMI, body cellular mass index; BMI, body mass index; FFM, fat-free mass; FFMI, fat-free mass index; FM, fat mass; FMI, fat mass index; PhA, phase angle. * The data are shown as mean (standard deviation).

**Table 3:** Dietary intake in newly diagnosed celiac patients (baseline and after two years on a gluten-free diet) and healthy controls.

	Celiac Patients Basal	Celiac Patients 2y GFD	Healthy Controls	*p*
Energy (kcal)	1697.3 (1381.8–1972.1)	1837.1 (1457.5–2193.0)	1858.4 (1622.8–2037.4)	0.889
Fat (g)	71.5 (50.0–82.1)	69.6 (60.7–91.9)	66.0 (47.8–76.6)	0.720
Protein (g)	64.2 (51.6–83.7)	77.8 (66.5–99.9)	75.7 (63.4–86.2)	0.596
Carbohydrates (g)	189.9 (134.6–222.1)	202.1 (166.6–222.7)	223.4 (172.9–245.2)	0.212
Sugar (g)	26.5 (13.3–51.5)	43.3 (29.0–47.45)	42.7 (27.3–51.1)	0.268
Fiber (g)	10.7 (9.3–14.2)	11.4 (8.6–17.8)	11.0 (8.3–15.5)	0.596

Data are given as median (interquartile range).

**Table 4:** Longitudinal changes in dietary intake in celiac patients at diagnosis (T0) and after 12 months on a gluten-free diet (T12).

	T0	T12	*p*
Energy (kcal)	1697.3 (1381.8–1972.1)	2076.4 (1672.0–2232.7)	0.126
Fat (g)	71.5 (50.0–82.1)	72.9 (60.5–96.7)	0.391
Protein (g)	64.2 (51.6–83.7)	89.9 (70.3–99.2)	0.296
Carbohydrates (g)	189.9 (134.6–222.1)	227.3 (194.2–255.2)	0.247
Sugar (g)	26.5 (13.3–51.5)	38.2 (26.3–55.2)	0.117
Fiber (g)	10.7 (9.3–14.2)	11.9 (9.2–14.4)	0.737

Data are given as median (interquartile range).

## Data Availability

The data presented in this study are available on request from the corresponding author. The request must be justified.

## Supplementary Materials

The following supporting information can be downloaded at: https://www.mdpi.com/article/10.3390/jcm14145061/s1. Table S1. Differences in body composition parameters in patients with celiac disease at the onset of the disease (T0) and after 12 months (T12) following a gluten free diet (*n* = 25). Table S2. Bioelectrical impedance analysis parameters at 12 months of GFD compared with healthy controls.

## Abbreviations

CD	celiac disease
GFD	gluten-free diet
BMI	body mass index
BIVA	bioelectrical impedance vector analysis
SF-BIA	phase-sensitive bioelectrical impedance analysis
R	resistance
Xc	reactance
PhA	phase angle
BCM	body cell mass
IQR	interquartile range
FM	fat mass
FFM	fat-free mass
FMI	fat mass index
FFMI	fat-free mass index
BCMI	body cellular mass index
