# Supplementary material for: Cellular Recovery and Body Composition Changes in Pediatric Celiac Disease After the Start of a Gluten-Free Diet: A Prospective Cohort Study
Source: J Clin Med. 2025 Jul 17;14(14):5061. doi: 10.3390/jcm14145061 (PMC12295783; doi:10.3390/jcm14145061)
Supplement: Supplementary file 1 [file jcm-14-05061-s001.zip › jcm-3741951-supplementary.pdf]

Supplementary tables

Supplementary Table S1. Differences in body composition parameters in patients with celiac disease at the onset of the disease (T0) and after 12 months (T12) following a gluten free diet (n = 25).

	T0	T12	p
**Anthropometry**			
Z score Weight	0.1 (-0.7 – 0.5)	0.02 (-0.6 – 0.6)	0.989
Z score Height	0 (-1.3 – 0.6)	-0.5 (-1.4-0.2)	**0.005**
Z score BMI	0.05 (-0.6-0.8)	0.2 (-0.1-1.1)	**0.026**
**Body Fat composition**			
FM (kg)	8.2 (6.6-13.9)	10.1 (6.9-16.6)	**0.001**
FMI (kg/m^2)	4.8 (4.1-7.6)	5.9 (4.6-7.7)	**0.023**
**Body lean mass composition**			
FFM (kg)	19.1 (15.6-29.6)	20.7 (17.1-31.6)	**0.001**
FFMI (kg/m^2)	11.6 (10.8-13.0)	11.9 (11.1-13.4)	0.397
BCM (kg)	9.4 (7.4-15.3)	10.4 (8.1-16.5)	**0.0001**
BCMI (kg/m^2)	5.7 (5.0-6.8)	6.0 (5.5-7.0)	0.059
**Muscle quality**			
PhA (degree)	5.0 (4.7-5.5)	5.3 (4.7-5.7)	0.121
Z score PhA	-1.2 (-2.1- -0.4)	-0.8 (-1.8- -0.1)	0.128
Na/K*	1.126 ± 0.1692	1.079 ± 0.1587	**0.015**

Data are given as median (interquartile range). BCM, body cellurar mass; BMI, body mass index; FFM, free fat mass; FFMI, fat mass index; FM, fat mass; FMI, fat mass index; PhA, phase angle. * The data are shown as mean (standard deviation).

Supplementary Table S2. Bioelectrical impedance analysis parameters at 12 months of GFD compared with healthy controls.

	Celiac patients 12m GFD (n=25)	Healthy controls (n=29)	p
**Age (years)**	10 (8-12)	9 (7.5-11.5)	0.452
**Females, n (%)**	13 (52)	17 (58.6)	0.587
**Anthropometry**			
Z score Weight	0.02 (-0.6 – 0.6)	-.02(-1.7 – 0.6)	0.567
Z score Height	-0.5 (-1.4-0.2)	0.08 (-1.4-0.7)	0.170
Z score BMI	0.2 (-0.1-1.1)	-0.2 (-1.2-0.7)	0.098
**Body Fat composition**			
FM (kg)	10.1 (6.9-16.6)	8.8 (6.3-11.6)	0.215
FMI (kg/m^2)	5.9 (4.6-7.7)	5.0 (3.8-5.9)	**0.05**
**Body lean mass composition**			
FFM (kg)	20.7 (17.1-31.6)	20.5 (16.6-25.7)	0.735
FFMI (kg/m^2)	11.9 (11.1-13.4)	11.5 (10.6-12.7)	0.241
BCM (kg)	10.4 (8.1-16.5)	9.7 (7.7-12.7)	0.515
BCMI (kg/m^2)	6.0 (5.5-7.0)	5.6 (5.0-6.1)	0.095
**Muscle quality**			
PhA (degree)	5.3 (4.7-5.7)	5.0 (4.7-5.2)	**0.026**
Z score PhA	-0.8 (-1.8- -0.1)	-1.3 (-1.9 - -0.8)	**0.049**
Na/K*	1.079 ± 0.1587	1.169 ± 0.1257	**0.035**

Data are given as median (interquartile range). BCM, body cellurar mass; BMI, body mass index; FFM, free fat mass; FFMI, fat mass index; FM, fat mass; FMI, fat mass index; PhA, phase angle. * The data are shown as mean (standard deviation).
